# Hybrid endovascular repair of aneurysmal right-sided aortic arch and Kommerell’s diverticulum using a two-vessel branched stent graft: Case report and review of literature

**DOI:** 10.1177/2050313X17749082

**Published:** 2017-12-21

**Authors:** Mo Hamady, Paritosh M Sharma, Radhika Patel, Anthony D Godfrey, Colin D Bicknell

**Affiliations:** 1 Department of Interventional Radiology, St Mary’s Hospital, Imperial College Healthcare NHS Trust, London, UK; 2 Department of Surgery and Cancer, Imperial College London, London, UK; 3 Imperial Vascular Unit, St Mary’s Hospital, Imperial College Healthcare NHS Trust, London, UK

**Keywords:** Thoracic aorta, aneurysm, branch stent graft, thoracic endovascular aortic repair, Kommerell’s diverticulum, right-sided arch

## Abstract

Right-sided aortic arches are rare, affecting approximately 0.1% of the population. They are a result of abnormal development of the primitive aortic arches and may present later in life with later life with aneurysmal expansion of the aberrant left subclavian artery ‘Kommerell’s diverticulum’. These can be challenging to treat effectively. We report a rare case presenting with mild dysphagia and right-sided aneurysmal aortic arch with aneurysmal aberrant left-sided. The patient underwent hybrid endovascular repair incorporating bilateral carotid–subclavian bypasses and dual-arch-branch endograft placement to the left and right common carotid arteries. Although endovascular approaches have been described, there are no reports of branched endografts in this scenario. Right-sided aneurysmal aortic arch and the aneurysmal aberrant left subclavian artery are rare and represent a significant therapeutic challenge. Endovascular repair in conjunction with extra-anatomical bypass utilising a custom-made branched thoracic endograft is feasible.

## Introduction

Right-sided aortic arches are rare, affecting approximately 0.1% of the population.^1^ They are a result of abnormal development of the primitive aortic arches. When associated with other congenital heart defects, they usually present in early life. However other variants, not associated with congenital heart defects, may go undiagnosed or may present in later life with aneurysmal change. These can be challenging to treat effectively. This article reports the treatment of an aneurysmal arch with a custom-made dual branch thoracic stent graft.

## Case

**Figure 1.**
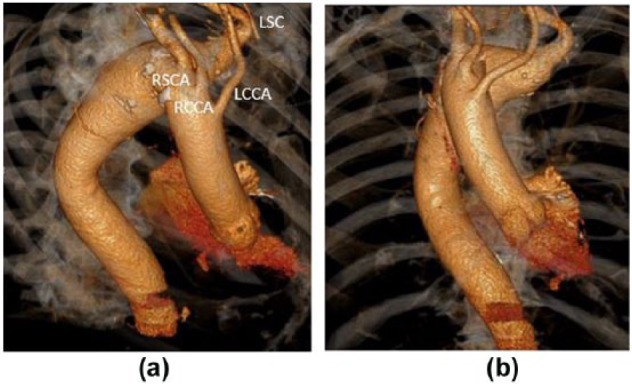
Pre-operative volume rendering CT angiogram imaging demonstrating: (a) a right-sided arch, aberrant origin of the left subclavian artery and (b) a Kommerel’s diverticulum at its origin. LSA: left subclavian artery; LCCA: left common carotid artery; RCCA: right common carotid artery; RSA: right subclavian artery.

We present the case of a 78-year-old male who had a few months history of mild dysphagia. He had been investigated at his local hospital with an oesophagoduodenoscopy (OGD), that had revealed mild extrinsic compression of the oesophagus and a computed tomography (CT) (Figure 1(a) and (b)) scan that revealed a right-sided arch with an aberrant left subclavian artery passing posterior to the oesophagus. A Kommerell’s diverticulum was identified with both the origin of the left subclavian artery and the aorta at that level being aneurysmal, measuring 4 and 5.2 cm, respectively. The CT scan measurements were taken in corrected plan to the centreline. His past medical history included paroxysmal atrial fibrillation, hypertension, hypothyroidism and hypercholesterolaemia. Pre-operative work up with echocardiogram; lung function tests did not reveal significant impairment. He refused an open repair with potential prolonged recovery, as he was the single carer of his disabled wife.

Following multi-disciplinary discussion and after full consent, the patient underwent a bespoke hybrid endovascular repair, incorporating bilateral carotid–subclavian bypasses and branched thoracic endograft placement (branches for both common carotid arteries). A dual branch custom-made endograft (Bolton Medical, Inc., Sunrise, FL, USA) was manufactured with the following features: proximal diameter of 40 mm, distal diameter of 36 mm, total length of 270 mm and tunnel length and diameter of 45 and 10 mm, respectively. Those measurements ensure 20% oversizing for the landing zones.

**Figure 2.**
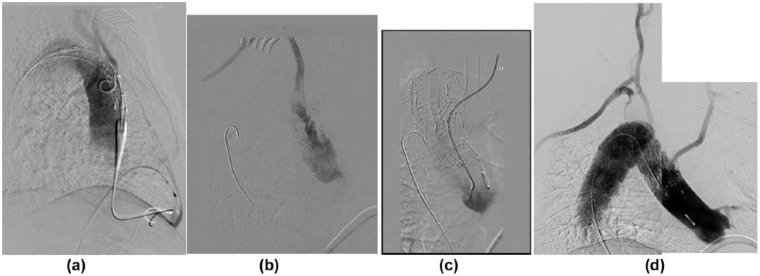
Intra-arterial DSA in right anterior oblique projection: (a) diagnostic angiogram prior to stent deployment, (b) after stent deployment and cannulation of the posterior tunnel (right common carotid artery), (c) after cannulation of the anterior tunnel (left common carotid artery) and (d) completion angiogram after deployment of bridging stent grafts.

The patient was positioned supine, sat up at 45°. Bilateral carotid–subclavian bypasses were fashioned sequentially through transverse supraclavicular incisions using 6-mm Dacron grafts. Both the left and right subclavian arteries were ligated proximal to the origin of the vertebral arteries. The justification of this bilateral bypass approach and ligation of native subclavian artery is the fact that stent graft implantation in the aortic arch, even with two branches for the two common carotid arteries, will cover the origin of both subclavian arteries including the antegrade flow to the vertebral arteries. Therefore, revascularisation of both subclavian arteries with preservation of flow to the vertebral arteries is strongly recommended to prevent posterior cerebral infarction. Right ventricular pacing lead inserted via right groin access was positioned and tested. The left common femoral artery (CFA) was accessed via a formal cut-down. The dual branch endograft was introduced over a stiff wire. Angiographic imaging was performed in the 30° right anterior oblique (RAO) projection for optimum visualisation of the origins of the carotid arteries, utilising an imaging catheter placed percutaneously through the right CFA. The endograft was then positioned in the thoracic aorta (Figure 2).

Rapid cardiac pacing was commenced (target 200 beats per minute), and cessation of cardiac output was confirmed. The graft was then deployed under fluoroscopic guidance before return of normal rhythm and cardiac function.

The cannulation of the two tunnels of the stent graft was expected to be challenging due to unusual anatomy and orientation of the great vessels. Therefore, meticulous pre-procedure planning using the CT scan identified the best image angulation and tube position that unravel the origin of the tunnels in relation to the carotid arteries. Right and the left common carotid artery approaches were utilised sequentially to cannulate the tunnels in thoracic endograft. This approach was chosen because the carotid retrograde approach had a straight angle. Lifestream^©^ (Bard Medical, Covington, GA, USA) (8 mm × 58 mm) balloon expandable covered stent was introduced first and followed by VIABHAN ^©^ (W.L. Gore & Associates, Inc., Newark, DE, USA) (13 mm × 50 mm) in each side and deployed in telescopic fashion. The left and right common carotid arteries measure 6.8 and 6.6 mm, respectively. The stent graft tunnels measure 10 mm each. It was the operator’s choice to use higher radial force balloon expandable covered stent first to strengthen the stented segment of the artery as it comes off the native aortic arch and courses through the thoracic inlet while leaving the lower profile covered stent to overlap with the tunnel and match up the needed oversizing.

No balloon expandable or self expandable bare stent was used to strengthen the Viabhan stent because the course of the stent was quite straight and not compromised. This is different from chimney technique where the bridging stent is competing with the main stent graft for space.

No kissing balloon was used in this case as the adjacent tunnels of the branch graft are close but separate from each other.

**Figure 3.**
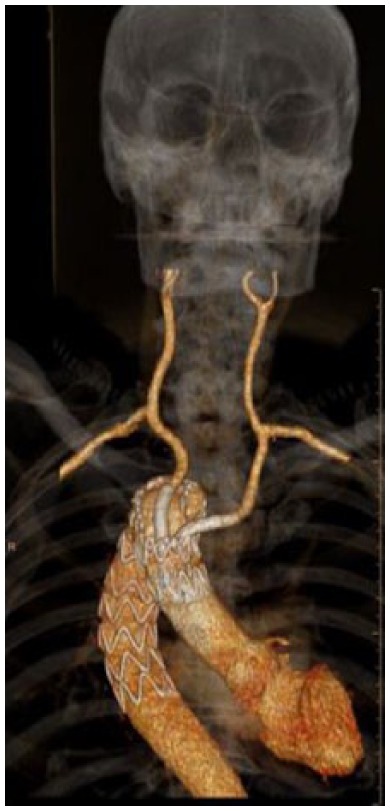
Three-dimensional volume rendering CT angiogram at 3 months.

Post-operatively, the patient was managed on the intensive care unit for 24 h and then in an intermediate care unit for another 48 h. His post-operative recovery was complicated with a chest infection that required treatment with intravenous antibiotics. A CT scan performed on post-operative day 5 revealed good position of the thoracic endograft and carotid stents (Figure 3) with no endoleak or graft kinking. He was subsequently discharged home on post-operative day 7. The patient demonstrated no clinical or radiological evidence of stroke. He remains well on 18-month follow-up with significant improvement in the presenting complain of mild dysphagia. The 18-months’ CT angiography that covers the head, neck and chest shows complete aneurysm seal, patent branches, patent vertebral arteries with intact circle of Willis and slight shrinkage in the Kommerell’s diverticulum and arch aneurysms, measuring 3.6 and 4.8 cm, respectively. There was no evidence of brain infarct.

Ethical approval was waived according to our institutional ethics committee rules.

## Discussion

A right-sided arch affects approximately 0.1% of the population. During normal embryogenesis, the left dorsal aorta persists and forms the normal left-sided arch. In patients with a right-sided arch, however, it is the right dorsal aorta that persists, the left dorsal aorta regresses. A right-sided arch can be associated with other variations in the course of the arch, supra-aortic branch anatomy and intra-cardiac defects.^2^ Several classification systems have been described, the two main types being the mirror image branching variety (Type I) and that associated with an aberrant left subclavian artery (Type II). The aberrant left subclavian artery in these cases may be associated with a Kommerell’s diverticulum – a retro-oesophageal aortic diverticulum at the origin of the artery. It is a remnant of the left dorsal aortic arch.

Patients with right-sided aortic arches may be asymptomatic. Those associated with other congenital heart defects may commonly present in childhood with symptoms related to these. Right-sided arches may also be associated with a vascular ring involving the trachea and oesophagus leading to compression of these structures.

In later life, patients may present with local pressure-related symptoms such as dysphagia, dyspnoea, oesophageal spasm, recurrent chest infections and chest pain as a consequence of a dissection or an aneurysmal change either of the aorta or at the Kommerell’s diverticulum.^3^ Rupture of these aneurysms is usually fatal due to the difficult access to the origin of an aberrant right subclavian artery especially when complicated by the presence of an aneurysm.^4,5^

Diagnosis of these patients may be confirmed with CT angiogram. These are essential to obtain an accurate assessment of the complex anatomy in these patients.

Management of such patients with right-sided arches and aneurysmal change is a difficult area fraught with the risk of developing significant complications. Successful open repair has been previously reported,^6–8^ however this mandates thoracotomy and frequently partial or complete cardio-pulmonary bypass with or without deep hypothermia with its incumbent perioperative morbidity and mortality. Endovascular approaches have been uncommonly described. Naoum et al.^9^ reported on a case of a right-sided arch and descending aorta with a large Kommerell’s aneurysm incorporating an aberrant left subclavian artery that was managed with a thoracic endograft and a carotid–subclavian bypass. Midorikawa et al.^10^ reported a case of a right-sided arch with an aberrant left subclavian artery that was successfully treated with a thoracic endograft and coil embolisation of the origin of the left subclavian artery without revascularisation. However, we are not aware of reports on hybrid arch repair using two-vessel thoracic branched stent graft.

## Conclusion

Right-sided arches associated with aneurysmal change and aberrant subclavian arteries are rare and represent a significant therapeutic challenge particularly due to the myriad of possible vascular anatomical configurations. Endovascular repair in conjunction with extra-anatomical bypass utilising a branched or fenestrated thoracic endograft is feasible after careful planning.
